# Physiological stress-induced corticosterone increases heme uptake via KLF4-HCP1 signaling pathway in hippocampus neurons

**DOI:** 10.1038/s41598-017-06058-6

**Published:** 2017-07-18

**Authors:** Hongxia Li, Caixia Zhang, Hui Shen, Zhilei Shen, Lusha Wu, Fengfeng Mo, Min Li

**Affiliations:** 1 Department of Ship Hygiene, Faculty of Naval Medicine, Second Military Medical University, Shanghai, 200433 China; 2 Department of Nursing, People’s Libration Army of 266 Hospital, Chengde City, Hubei 067000 China

## Abstract

Iron overload has attracted much attention because of its adverse effect in increasing the risk of developing several neurodegenerative disorders. Under various pathologic conditions, a lot of heme are released. The aggregation of heme is more neurotoxic than that of iron released from the heme breakdown. Our previous studies demonstrated that psychological stress (PS) is a risk factor of cerebral iron metabolism disorders, thus causing iron accumulation in rat brains. In the present study, we found PS could increase heme uptake via heme carrier protein 1 (HCP1) in rat brains. We demonstrated that Glucocorticoid (GC), which is largely secreted under stress, could up-regulate HCP1 expression, thus promoting heme uptake in neurons. We also ascertained that HCP1 expression can be induced by GC through a transcription factor, Krüppel-like factor 4 (KLF4). These results may gain new insights into the etiology of heme uptake and iron accumulation in PS rats, and find new therapeutic targets of iron accumulation in Parkinson’s disease or Alzheimer’s disease.

## Introduction

Heme, a ferrous protoporphyrin IX that serves as the major source of iron in the body, plays a pivotal role in iron metabolism and a myriad of other cellular processes, including electron transport, gas synthesis, gas sensing, signal transduction, microRNA processing, and maintenance of the circadian clock^1–3^. However, an excessive amount of free heme promotes lipid peroxidation and increases oxidative stress through generation of reactive oxygen species (ROS). Such a process may give rise to membrane injury, ultimately resulting in cell apoptosis^4^. Therefore, the intracellular levels of free heme must be tightly regulated^5^.

Under normal physiological conditions, free heme is rarely detected, thus concentrations are expected to be negligible. However, under various pathologic conditions, such as hemorrhage, hemolysis or cell injury, large amounts of heme are released and contribute to brain damage^6–8^. Such an excess in free heme can lead to other physiological disorders, such as complications after hemorrhagic stroke^9–11^. Previous studies demonstrated that the incidence of brain microhemorrhages increases with age^12, 13^ and contributes to Alzheimer disease pathology^14, 15^. Recent studies suggested that the accumulation of heme is more neurotoxic than that of iron released from the heme breakdown^16^. Hemin is the oxidized form of heme, and though the neurotoxic effect of hemin has been previously demonstrated in primary neuronal cultures and neuronal cell lines^11, 17^, the underlying mechanisms of hemin uptake by brain cells remains largely unclear.

Heme carrier protein 1 (HCP1), also known as proton-coupled folate transporter (PCFT)^18^, is a membrane transporter belonging to the major facilitator superfamily. It was first identified and described as being able to take up heme in the duodenum^19^. Subsequent research demonstrated that HCP1 could also mediate heme uptake in the retina, retinal pigment epithelium, and astrocytes^20, 21^. 5′-aminolevulinate synthase 1 (ALAS1) catalyzes the first step in the heme synthesis, while heme oxygenase 1 (HO-1) catalyzes the step in heme degradation. ALAS1 and HO-1 are the rate-limiting enzymes in heme biosynthesis and catabolism, respectively^22^. Previously we have reported that psychological stress (PS) could induce dysregulation of iron metabolism^23, 24^. PS significantly decreases serum iron and affect erythropoiesis. In PS rats, iron absorption decreased and iron is significantly accumulated in the apical poles of villous enterocytes^25^. We also have demonstrated that PS could increase iron intake and cause iron accumulation in liver^23, 26^ as well as in most regions of the rat brain^27, 28^. Our data suggested that PS was a risk factor of cerebral iron metabolism disorders. Heme, as the major source of iron, plays a pivotal part in iron metabolism. However, the role of HCP1 in heme uptake in the brain under PS condition is still unclear and has never been reported. Glucocorticoid (GC) is known to regulate intestinal expression of heavy and light ferritin and can be induced by PS^29^. We previously demonstrated that GC could up-regulate the expression of iron regulatory protein 1(IRP1) in the liver. In addition, HCP1 protein could also be induced by GC in macrophages^30^.

The aim of this study is to investigate the influence of PS on hemin uptake in the rat brain. We examined the iron content in the cerebral cortex and hippocampus of the PS rat brain, and tested for stress hormones in blood. In addition, we determined the direct effect of corticosterone on HCP1 expression and its promotion on hemin uptake in mouse hippocampal cells (HT-22). We also demonstrated that the effect of corticosterone on HCP1 is through the transcription factor of Krüppel-like factor 4 (KLF4).

Our studies help understand the effect of corticosterone on hemin uptake in the brain, gain new insights into the etiology of iron accumulation in PS and may also lead to new therapeutic targets to treat iron accumulation in Parkinson’s disease or Alzheimer’s disease.

## Results

### GC increases following PS treatment could be associated with enhanced HCP1, KLF4 and HO-1 expression in the hippocampus and cortex of rats

**Figure 1:**
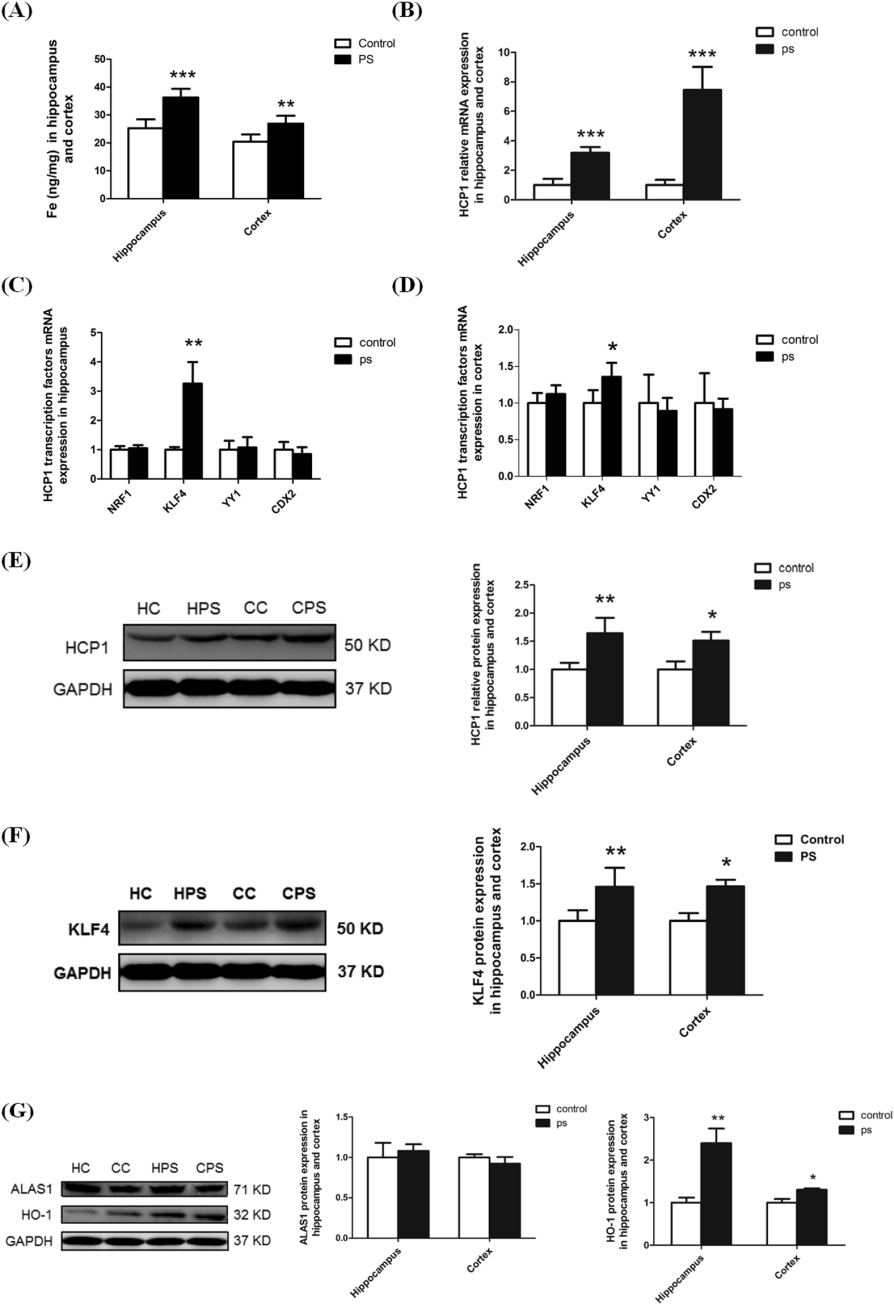
Iron content, HCP1 and its transcription factors expression in hippocampus and cortex of rat brain. (**A**) The iron content in hippocampus and cortex was significantly elevated in PS group compared with the control group. (**B**) RT-PCR analysis showed that HCP1 mRNA expression in hippocampus and cortex of PS rat brain was significantly higher than in control group. (**C**,**D**) The transcription factors NRF1, KLF4, YY1 and CDX2 mRNA expression in hippocampus and cortex of the rat brain: only KLF4 mRNA expression was significantly increased in PS group, and others have no significant differences compared with the control group. (**E**) Western blot analysis revealed that HCP1 protein expression was increased in hippocampus and cortex of the PS rat brain. (**F**) Western blot analysis also demonstrated that KLF4 protein expression was increased in hippocampus and cortex of the PS rat brain. (**G**) Western blot showed that ALAS1 expression in hippocampus and cortex of PS rat brain has no differences with the control group, while the expression of HO-1 was significantly increased in PS group. Values are means ± SD. *p < 0.05; **p < 0.01; ***p < 0.001. (HC: hippocampus in control group; HPS: hippocampus in PS group; CC: cortex in control group; CPS: cortex in PS group).

After 7-days PS exposure, rats were euthanized. To validate our PS rat model, levels of serum corticosterone (CORT), Adrenocorticotropic Hormone (ACTH) and Norepinephrine (NE) were tested. As shown in Table 1, the serum levels of CORT, ACTH and NE in the PS group were significantly higher than those in the control group, which are consistent with our^31^ and others’^32, 33^ previous reports. Next, iron content in cerebral cortex and hippocampus was detected by atomic absorption. The result showed that the iron content in PS rats is significantly greater than that in control rats (*p* = 0.000258 in hippocampus and *p* = 0.002304 in cortex, Fig. 1A). Since total iron content can’t distinguish heme and non-heme iron, RT-PCR assays were performed to detect the HCP1 expression in cerebral cortex and hippocampus. The results, shown in Fig. 1B, indicated that HCP1 transcripts are significantly higher in PS rats than that in control rats (*p* = 0.0000733 in hippocampus and *p* = 0.00000429 in cortex). It has been reported that KLF4, NRF1, YY1, and CDX2 and HNF4α are potential transcription factors of HCP1^34, 35^. To investigate the potential mechanism of enhanced expression of HCP1 in PS rat brain, we also performed RT-PCR analysis for the expression of the above HCP1 transcription factors in the rat brains. It was evident that only KLF4 mRNA expression was significantly increased in the cerebral cortex and hippocampus of the PS rat brains (*p* = 0.006594 in hippocampus and *p* = 0.0377 in cortex), while NRF1, YY1, and CDX2 expression have no significant difference with the control group (Fig. 1C and D). Western blot assays were then performed to: i) indicate protein expression of tissue specific markers; and ii) confirm the significantly enhanced HCP1 and KLF4 expression at protein level in the cerebral cortex and hippocampus of PS rat brain (Fig. 1E and F). In addition, we also tested the expression of ALAS1 and HO-1. The results indicated that the expression of ALAS1 in PS rat brain has no significant differences with that of the control group, while the expression of HO-1 in PS rat brains was significantly increased (*p* = 0.005736 in hippocampus and *p* = 0.0157 in cortex Fig. 1G). Taken together, our results suggested that the enhanced iron level in PC rat brain is, at least partially, contributed by HCP1 pathway.

**Table 1:** Serum concentrations of CORT, ACTH and NE levels in rats of PS and control groups.

	CORT (ng/ml)	ACTH (ng/ml)	NE (ng/L)
Control group	355.05 ± 35.53	330.25 ± 33.38	52.74 ± 11.45
PS group	426.13 ± 47.64**	393.56 ± 36.51*	89.61 ± 18.38*

Values are means ± S.D, n = 10. **p < 0.01; *p < 0.05 vs control group.

### HCP1 plays an essential role in heme uptake *in vitro*

**Figure 2:**
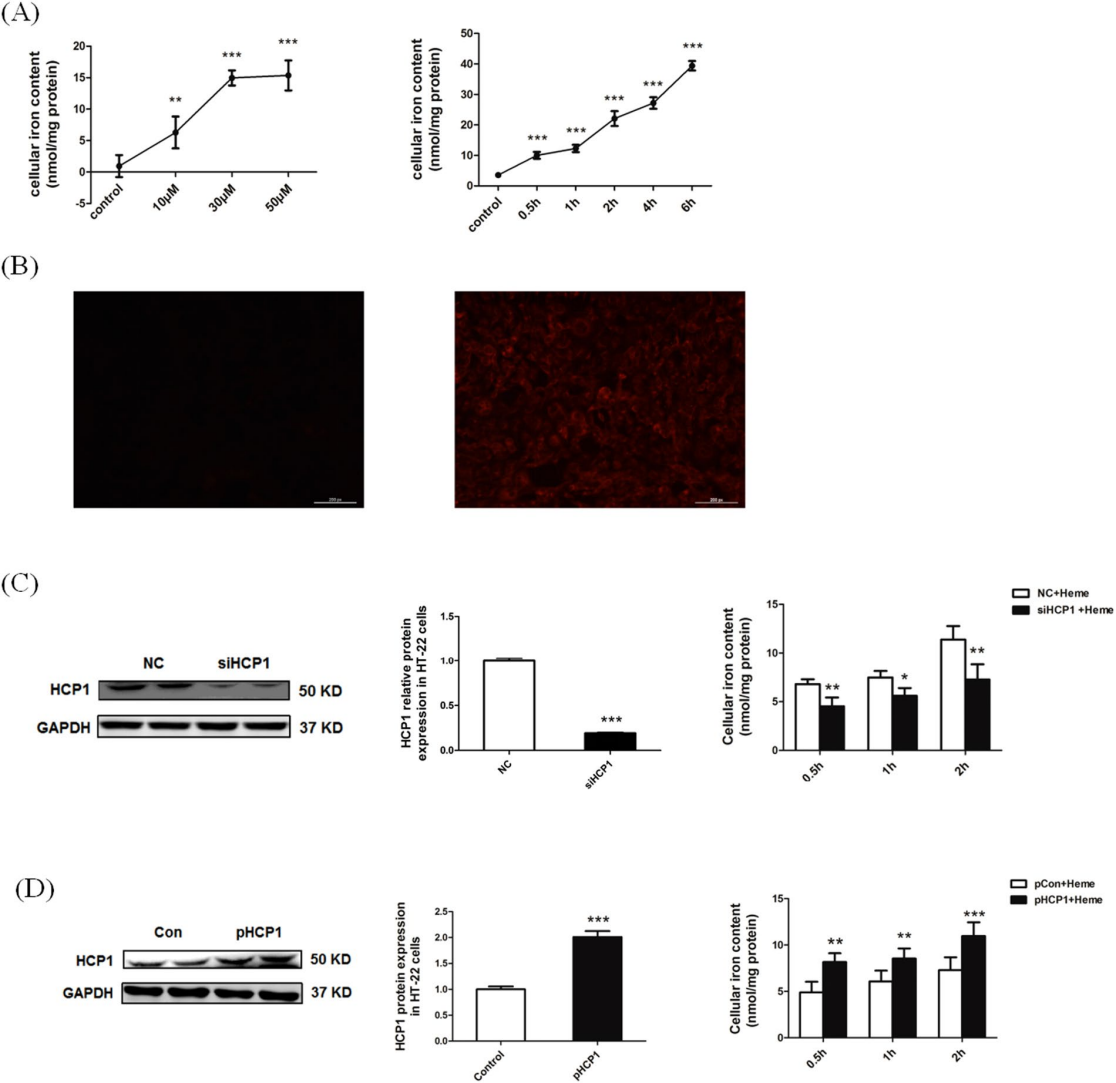
Mouse hippocampus nerve cells have the capacity to uptake heme via HCP1. (**A**) Iron content in cells treated with different concentrations of heme for 2 h and with 30 μM heme for different time. (**B**) To visualize hemin accumulation in neurons, cells were incubated with 30 µM ZnPPIX for 2 h, and the fluorescence in ZnPPIX treated cells was apparently intensified and compared with the control. (**C**)The expression of HCP1 was decreased when cells were transfected with HCP1 siRNA, and the uptake of heme was correspondingly decreased. (**D**) The expression of HCP1 was increased when cells were transfected with HCP1 expression plasmid, and the uptake of heme was correspondingly increased. Values are means ± SD. *p < 0.05; **p < 0.01; ***p < 0.001.

To investigate the role of HCP1 in heme uptake by hippocampus nerve cells, HCP1 siRNA was used to knockdown HCP1 expression in HT-22 cells 2 days prior to the treatment with hemin. It was found that the intracellular iron content was increased in both concentration and time gradient when cells were incubated with hemin (*p* < 0.01, Fig. 2A). To visualize hemin accumulation in neurons, cells were incubated with 30 µM ZnPPIX, which has been previously reported to be accumulated in cells via HCP1^19^. After 2 h incubation with ZnPPIX, strong cellular fluorescence was observed (Fig. 2B).

Western blot assays indicated that the HCP1 expression was significantly inhibited 48 hours post-transfection. When compared to a non-specific control siRNA (*p* < 0.0001), meanwhile, there was a significant decreased heme uptake upon HCP1 knockdown (Fig. 2C). In contrast, when an HCP1 expression plasmid was transfected into HT-22 cells, a significant enhanced heme uptake was observed (Fig. 2D).

### GC enhances HCP1 expression *in vitro*

**Figure 3:**
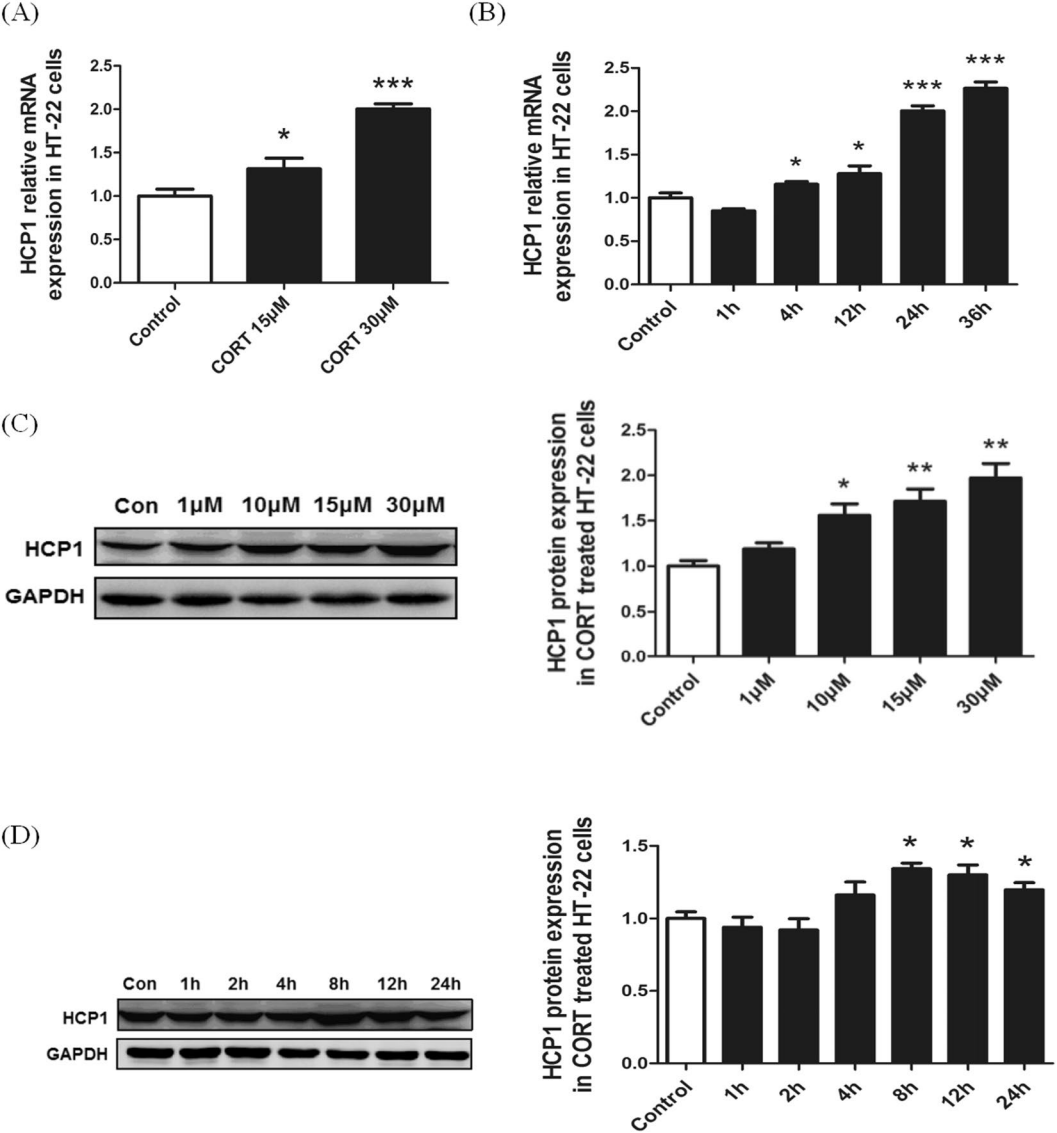
Corticosterone up-regulates HCP1 in HT-22 cells. (**A**) RT-PCR analysis showed that HCP1 mRNA expression was elevated when HT-22 was incubated with 15 μM and 30 μM CORT for 24 h. (**B**) HCP1 mRNA expression in HT-22 cells treated with 30 μM CORT for different times, and the HCP1 expression was increased after incubated for 4 h. (**C**) HCP1 protein expression in cells treated with different concentrations of corticosterone for 24 h, and the expression was increased when cells were treated with 10 μM corticosterone above. (**D**) HCP1 protein expression in cells treated with 30 μM corticosterone for different times, and the expression was increased after an 8 h incubation period. Values are means ± SD. *p < 0.05; **p < 0.01; ***p < 0.001.

It was previously shown that corticosterone is largely secreted under stress^36^. We also reported that increased extracellular corticosterone concentrations could cause iron accumulation in hippocampal neurons *in vitro* ^37^. We hypothesized that HCP1-mediated hemin uptake might be one of the mechanisms of corticosterone-induced iron accumulation in hippocampus neurons. To this end, HT-22 cells were treated with corticosterone and HCP1 expression was determined by both reverse-transcribed PCR (RT-PCR) and Western blot assays for mRNA and protein, respectively. Previous reports indicated that corticosterone impairs HT-22 cell viability at a concentration higher than 50 µM^38, 39^. Our results indicated that at as low as 15 µM, corticosterone treatment for 24 hours readily induced HCP1 mRNA transcription in HT-22 cells (Fig. 3A).The enhancement was more significant when corticosterone was used at 30 µM(Fig. 3A). In addition, the effect of corticosterone on HCP1 mRNA transcription is time-dependent (Fig. 3B). Similarly, Western blot analysis revealed that HCP1 protein expression was increased upon corticosterone treatment in a concentration- and time-dependent manner (Fig. 3C and D).

### GC enhances KLF4 expression and hemin uptake *in vitro*

**Figure 4:**
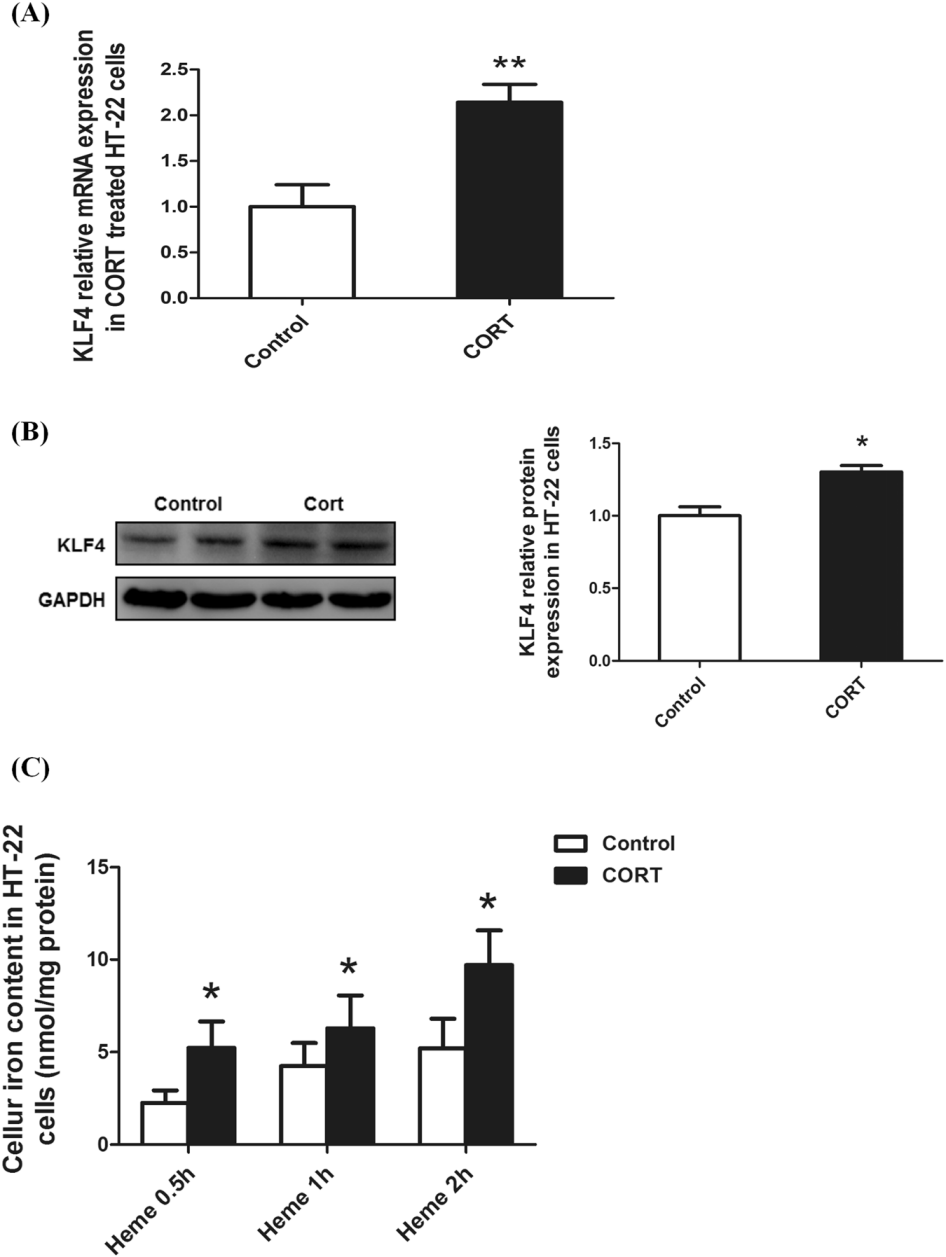
KLF4 expression and hemin uptake are increased in hippocampus and cortex of the PS rat brain and CORT-treated HT-22 cells. (**A**,**B**) KLF4 mRNA and protein expressions were elevated in HT-22 cells treated with 30 μM CORT for 24 h. (**C**) The cellular iron content was increased markedly in cells pre-treated with corticosterone than only treated with hemin. Values are means ± SD. *p < 0.05; **p < 0.01.

Next, the expression of KLF4, a potential transcription factor of HCP1, was determined upon corticosterone treatment at 30 µM for 24 hours. As shown in Fig. 4A, corticosterone treatment led to a significant increase (>2-fold) in the KLF4 mRNA expression in HT-22 cells (*p* = 0.0048). Meanwhile, Western blot analysis revealed that KLF4 protein expression was significantly increased upon corticosterone treatment (*p* = 0.0155, Fig. 4B). To investigate the effect of GC on heme uptake, cells were pre-incubated with corticosterone for 1 h, and then added hemin for 2 h. Interestingly,in the corticosterone and heme co-treated cells, the cellular iron content was increased more markedly than that in the only hemin treated cell (*p* = 0.017066, Fig. 4C).

### GC stimulates HCP1-meidated heme uptake *in vitro* through the activation of KLF4

**Figure 5:**
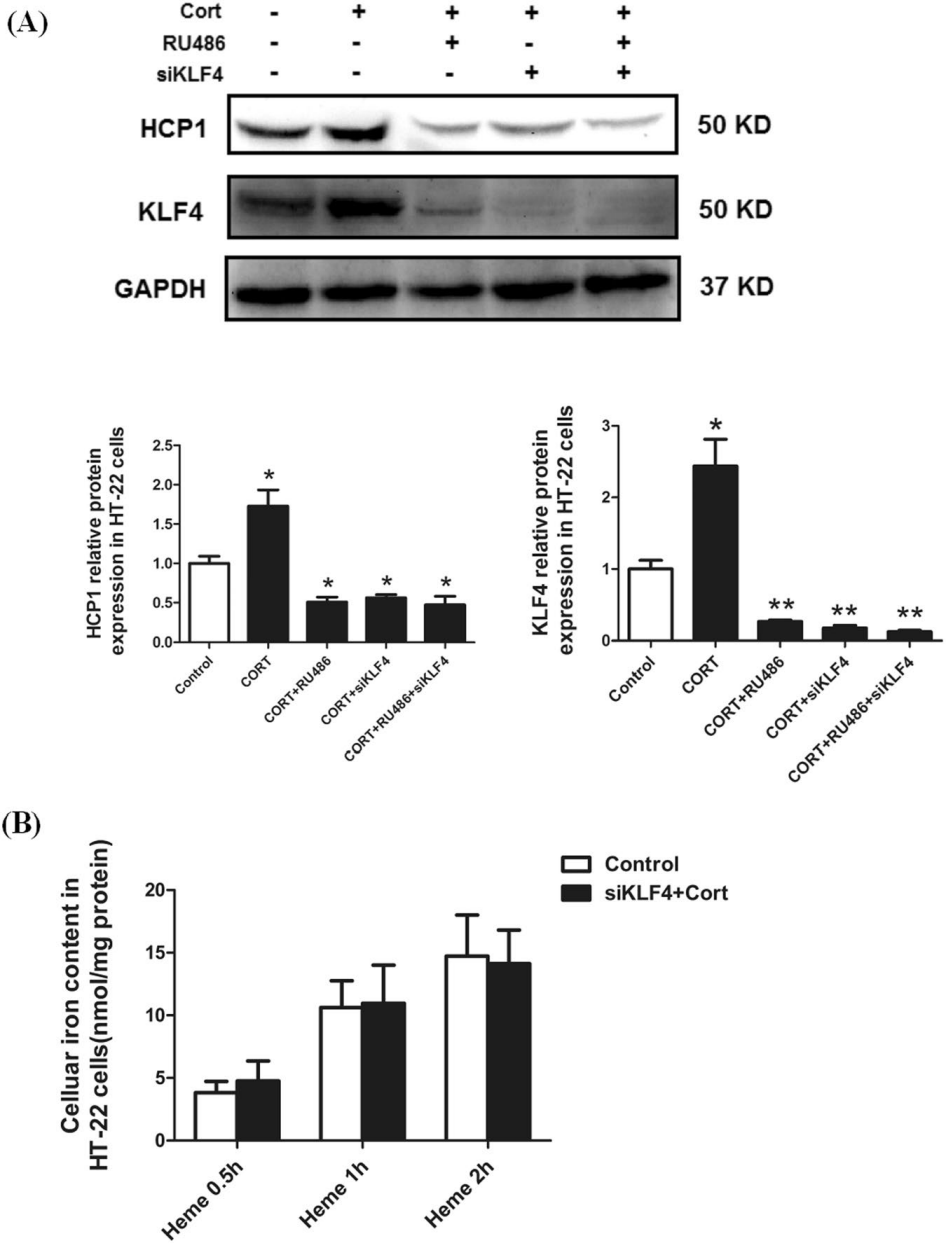
CORT stimulates HCP1 expression through activation of KLF4. (**A**) Western blot showed that HCP1 expression was increased when cells were treated with CORT. However, this effect was abolished when cells were pre-transfected with KLF4 specific siRNA or pre-incubated with the inhibitor of CORT (RU486). (**B**) HT-22 cells were pre-transfected with KLF4 siRNA, then treat with CORT and incubated with hemin. The cellular iron content originally increased by CORT was decreased, and has no differences with the control group. Values are means ± SD. *p < 0.05; **p < 0.01.

To investigate the role of KLF4 in the effect of corticosterone on HCP1-mediated heme uptake, HT-22 cells were treated with KLF4 specific siRNA and the inhibitor of GC receptor (RU486). The present results showed that the increased HCP1 expression induced by corticosterone was significantly reduced when cells were pretreated with RU486 or transfected with KLF4 siRNA (*p* < 0.05, Fig. 5A). Moreover, the effect of GC on heme uptake in HT-22 cells was also abolished when cells were transfected with KLF4 siRNA (Fig. 5B). These indicate that the expression of HCP1 and increased heme uptake induced by GC in HT-22 cells or PS rat brain may be through KLF4.

## Discussion

Our previous studies demonstrated that PS could cause iron accumulation in the cerebral cortex and hippocampus of the rat brain^27, 28^, and suggested that PS is a risk factor of disorders related to cerebral iron metabolism. Oxidative damage could be induced by increased iron^40^. In the present study, we demonstrated that in PS rat brains the expression of HCP1 could be induced by corticosterone through KLF4, and the enhanced HCP1 may increase heme uptake, which may partly lead to iron accumulation in the cerebral cortex and hippocampus, thus exacerbate oxidative damage in rat brain.

The iron content in different parts of brain varies greatly. Previously, we demonstrated that after PS exposure, the iron concentrations are increased in some specific regions of rat brain, which may be attributed to varying iron regulation factors. PA Dennery found that reactive iron can induce HO-1 expression^41^. In this study, we found the expression of HCP1 was significantly increased and accompanied by elevated concentrations of iron in the cerebral cortex and hippocampus of PS rat brains. We also found that increased expression of HO-1 in PS rat brain that can be induced by heme imported by HCP1. ALAS1 expression in PS rat brain has no significant differences compared with the control group. The elevated HCP1 means that much heme has been imported to cells^42^. Above results indirectly indicate that heme release is increased by PS and then heme is transported into brain cells by HCP1, raising the iron concentration in cerebral cortex and hippocampus of rat brain.

Previous studies have demonstrated that cultured astrocytes and cerebellar granule cells have the capacity to uptake hemin via HCP1^16, 21^, and cause iron accumulation in cells. In the present study we ascertained that hippocampal neurons can take up hemin by HCP1, and accumulate it in a time-and concentration-dependent manner. However, the neurotoxicity of heme to HT-22 cells seems more alleviated. The differences in hemin toxicity between the present study and Regan’s may be due to cell type differences^43^. The current study used immortalized cells whereas they used primary cells. Immortalized cells were used because the accumulation of hemin should be examined for up to 6 h without cell death.

To investigate the possibility that the neurons uptake hemin via HCP1, we used ZnPPIX (has autofluorescent property), which has previously been shown to accumulate in cells via HCP1^19^. The result showed that the bright fluorescence indicator appeared in the cells incubated with 40 µM ZnPPIX for 2 h. Furthermore, HCP1 expression was induced and inhibited by constructing the corresponding over-expression and siRNA plasmid. The present study demonstrated that when HCP1 expression was suppressed, the iron content in cells was decreased, and when HCP1 expression was enhanced, the iron content in cells was increased. The result further confirmed hemin could be taken up by HCP1 in hippocampal neurons.

Glucocorticoid is one of the most widely used anti-inflammatory agents in clinical practice, and is largely secreted under stress. The present study showed that serum corticosterone was increased markedly in PS rats. A study on macrophages reported that HCP1 could be induced by GC, and has an essential role in the regulation of Hb/heme-iron recycling^44^. Interestingly, the present study found that HCP1 expression was significantly increased in cerebral cortex and hippocampus of the PS rat brain. Furthermore, our study showed that HCP1 expression was increased in a concentration- and time-dependent manner when cells were treated with corticosterone. More important, the present study suggested cells treated with corticosterone accumulated more iron than the control. This illustrated that corticosterone could enhance HCP1 expression and therefore increase hemin uptake in neurons.

KLF4 (Krüppel-like factor 4), a member of the family of zinc-finger transcription factors, has diverse functions and is associated with a variety of pathophysiological processes. KLF4 is believed to be critical to endothelial and macrophage-mediated inflammation^45–48^, and is also a key factor in mediating neuroinflammation via microglial activation and the subsequent release of proinflammatory cytokines^49, 50^. Recent studies suggested that KLF4 may be involved in the regulation of reaction after ischemic injury in astroglial^51^, and play a key role in regulating the immunomodulatory activities of microglia^52^. Previous studies demonstrated that KLF4, NRF1, YY1, and CDX2 and HNF4αare potential transcription factors of HCP1/PCFT/SLC46a1^34, 35^. In the study, we detected the expression of above HCP1 transcription factors in the brains of rats, and found that only KLF4 expression was greatly increased in the cerebral cortex and hippocampus of PS rats. More importantly, our study suggested KLF4 expression is significantly elevated in corticosterone treated cells. To ascertain if corticosterone up- regulates HCP1 expression through KLF4, the glucorticoid-receptor inhibitor RU486 and KLF4 specific siRNA were administrated^26^. Western analysis showed a more than twofold increase in KLF4 expression in corticosterone treated cells. However, the RU486 and KLF4 silence RNA added significantly repressed the up-regulation of corticosterone on HCP1. Further, we demonstrated that the effect of corticosterone on hemin uptake via HCP1 was abolished when cells were transfected with KLF4 siRNA.Taken together we ascertain that through activated KLF4, corticosterone up-regulated HCP1, and resulted in increased hemin uptake in hippocampal neurons.

In our previous and this study, we clarified that the iron content increased in the hippocampus and cortex and there were many iron particles in the neurons in stress-exposed rats, however we can’t distinguish non-heme iron and heme iron. We can only speculate that the iron content in the physiological stress-exposed rats increased partly due to the strengthen of transporting heme, according to the elevated HCP1 and HO-1 indirectly. We think we should repeat this experiment in more cells such as neuroglia in our future experiment.

## Conclusion

Our study demonstrated that the expression of HCP1 was significantly increased in the cerebral cortex and hippocampus of the PS rat brain, and that CORT, which is largely secreted under stress, could greatly stimulate HCP1 expression and increase the uptake of hemin in hippocampal neurons. In addition, CORT stimulated HCP1 expression through the activation of the transcriptionfactor KLF4. Based on these findings, we postulate that CORT-induced elevation of HCP1 through KLF4 may be one of the reasons why PS can cause iron accumulate in the rat brain.

## Methods

### Animals and establishment of the PS model

Thirty male Sprague-Dawley (SD) rats, weighing 120 ± 5 g, were purchased from Shanghai-BK Co., Ltd., Shanghai, China, and were housed individually with free access to food and water. After 7-day of adaptation, rats were equally divided into three groups: control, PS, and foot shock (FS) groups. The PS model was created in rats by a communication box as described previously. Rats were exposed to PS for 30 min every morning (10:00–10:30) for 7 days. All animal procedures were performed strictly in accordance with the international ethical guidelines and approved by the animal research committee of the Second Military Medical University (Shanghai, China).

### Sampling preparation

At the end of PS exposure, animals were anesthetized by *i.p*. injection of 10% chloral hydrate. Blood samples were collected from the heart, and centrifuged at 3000 g for 20 min. The serum content of corticosterone (CORT), adrenocorticotropic hormone (ACTH), and norepinephrine (NE) were analyzed to evaluate the PS model using a commercially available ELISA kit (R&D Systems, Inc., USA).

Then, rats were perfused through the left cardiac ventricle with ice-cold phosphate buffered saline (PBS; pH 7.4) to flush out the blood. The cerebral cortex and hippocampus were quickly removed, snap frozen in liquid nitrogen, and kept at −80 °C for further assays.

### *In vitro* experiments with HT-22 cells

Mouse hippocampus nerve cells (Cell line HT-22, gifted by David Schubert, Salk Institute for Biological Studies, California) were grown in high-glucose DMEM supplemented with 10% FBS (Gibco, USA) and 1% antibiotic solution, and incubated in a humidified 5% CO_2_ atmosphere at 37 °C. The culture medium was replaced every 2–3 days.

### *In vitro* heme uptake assays

The cells were incubated with the medium containing 0, 10, 30 and 50 μM hemin (Sigma) for 2 h, and with 30 μM hemin for 0, 0.5, 1, 2, 4 and 6 h. After incubation, the cellular content of iron was quantified using an atomic absorption spectrophotometer as described previously^53^, and standardized against basal protein levels^21^.

To determine whether HCP1 contributes to heme uptake in neurons, we constructed a HCP1 expression plasmid and synthesized HCP1 siRNA. To visualize heme uptake via HCP1 in HT-22 cells, we used ZnPPIX (has autofluorescent properties), which has previously been shown to accumulate in cells via HCP1^19^.

### Corticosterone treatment

HT-22 cells were treated with 1 µM, 10 µM, 15 µM and 30 µM corticosterone for 24 h, and treated with 30 µM corticosterone for 1 h, 2 h, 4 h, 8 h, 12 h and 24 h. QPCR and western blot were used for investigating the effect of corticosterone on HCP1 expression. To ascertainthe effect of corticosterone on heme uptake, HT-22 cells were pretreated with corticosterone for 1 h, and then added 30 μM heme for 2 h. The cellular content of iron was quantified using an atomic absorption spectrophotometer as previously described^53^.

### siRNA silence of KLF4

HT-22 cells were transferred to 6-well plates and transfected with siRNA products for KLF4 silence or negative control oligos (Jima, China) in the presence of lipofectamine RNAiMAX according to the manufacturer’s instructions (Invitrogen, USA). Cells were maintained for 24 h after transfection and given 30 μM corticosterone for another 24 h. For testing the effect of corticosterone on KLF4 expression, RU486, an inhibitor of GR, was added to cells at 20 μM 1 h prior to the treatment with corticosterone at 30 μM.

### Real-time quantitative PCR analysis

Total RNA was extracted using Trizol (Invitrogen, USA) and reversely transcribed to cDNA by RT reagent Kit (Primerscript^TM^, TAKARA, Japan). Quantitative PCR amplification was performed with Real Time PCR Master Mix (TOYOBO Biotech Co., Ltd.) using StepOne Plus (ABI, USA). Primers were as follows. Rat and mouse HCP1 primer, sense: 5-CGCCATCACCGATCCATTGTCC-3, antisense: 5-AAAAGAGAGCACCCTGCTCCGA-3; KLF4 primer, sense: 5-CATCAGTGTTAGCAAAGGAAGC-3, antisense: 5-GTGGCATGAG CTCTTGATAATG-3.

### Western blotting

Homogenates of the rat cerebral cortex and hippocampus or HT-22 cell lysates were prepared for Western-Blot analysis. Proteins were incubated overnight at 4 °C with a primary antibody against HCP1 (1:1000, ab25134, Abcam, USA), ALAS1 (1:1000, AB21560, Sangon, China), HO-1 (1:500, ab13248 Abcam, USA), KLF4 (1:250, ab72543, Abcam, USA), and GAPDH (1: 2000, Cell signal technology, USA). The blots were developed by incubation in ECL chemiluminescence reagent (Amersham Life Science, Arlington Heights, IL, USA) and subsequently exposed to BioMax Light Film (Eastman Kodak Co., USA).

### Statistical analysis

The values are presented as mean ± SD. Statistical analysis was carried out using SPSS 16.0 (SPSS, Inc., Chicago, IL, USA). Statistical difference between two groups was assessed by the Independent-t test. One way ANOVA, followed by LSD-t and SNK post-hoc test, were performed to analyze the difference between the three or more groups. Differences were considered statistically significant at p < 0.05.
